# Remission with Cabergoline in Adolescent Boys with Cushing’s Disease

**DOI:** 10.4274/Jcrpe.1007

**Published:** 2013-09-18

**Authors:** Ayla Güven, Feyyaz Baltacıoğlu, Fatma Dursun, Ayşe Nurcan Cebeci, Heves Kırmızıbekmez

**Affiliations:** 1 Göztepe Education and Research Hospital, Pediatric Endocrinology Clinic, İstanbul, Turkey; 2 Marmara University, Medical Faculty, Department of Radiology , İstanbul, Turkey

**Keywords:** Cushing’s disease, cabergoline, adolescent, inferior petrosal sinus sampling

## Abstract

Cabergoline is a long-acting dopamine receptor agonist used for treatment of patients with uncured Cushing’s disease (CD) and, as a first-line treatment, was used in only limited numbers of patients. This report presents two adolescent boys with CD who were treated with cabergoline. Two adolescent boys with clinical and laboratory findings of CD are presented. No pituitary adenoma was detected by radiological investigation in either patient. Adrenocorticotropic hormone (ACTH) hypersecretion and lateralization was found by inferior petrosal sinus sampling in both patients. The initial cabergoline dose was 1mg/week and was adjusted up to 1.5 mg/week in the second patient, based on his urinary free cortisol (UFC) level. The patients responded to cabergoline treatment with normal UFC levels on the 4th and 6th months of treatment. The boys reached complete remission at the end of the 17th and 24th months, respectively. Cabergoline is effective in the control of cortisol secretion and can be considered as a first-line treatment in cases of CD.

**Conflict of interest:**None declared.

## INTRODUCTION

Cushing’s disease (CD) caused by a corticotrophic pituitary tumor is the most common form of Cushing’s syndrome (1,2,3). Transsphenoidal surgery is the first-choice treatment for most patients with CD. Surgery is effective in inducing immediate disease remission in around 70% and late disease remission in around 50% of patients (4,5,6). Pituitary irradiation and bilateral adrenalectomy are the alternative therapeutic approaches to CD, but these interventions can be associated with severe complications (7).

There is still a strong need for a medication option in the management of CD. Medication treatment usually consists of use of adrenal-blocking drugs and neuromodulatory drugs acting at the pituitary level (8,9). However, inhibitors of steroidogenesis and the adrenolytic agent mitotane have limited efficacy or cause side effects that restrain their long-term utilization (5). Studies on using dopamine agonists like bromocriptine or cabergoline were started following the demonstration of D2 receptor expression in corticotroph tumors (10). The effect of these drugs on adrenocorticotropic hormone (ACTH) and cortisol secretion was reported in a few in vitro and in vivo studies (10,11). A higher affinity and specificity of cabergoline for D2 receptors in addition to its longer half-life (12,13) could explain its better efficacy in CD. Reports of successful outcome range from 25 to 75% in short-course treatment of persistent or recurrent CD (14,15). However, data pertaining to cabergoline as a first-line therapy in cases of CD are scarce. Experience with cabergoline in childhood and adolescence is also limited (16). Here, we describe two boys with CD (17 and 15 years old), in whom first-line cabergoline treatment was effective in inducing remission.

## CASE REPORT

**Patient 1:** A 17-year-old male who presented with weight gain and hypertension was found to show the clinical features of CD including obesity, pink striae, and acanthosis nigricans. His weight was 92 kg, height 175 cm. Body mass index (BMI) was 30 kg/m2, blood pressure 140/100 mmHg, and pubertal stage was Tanner V. Clinical features and hormonal workup are shown in Table 1. Investigations revealed findings suggestive of ACTH-dependent CD, as follows: a basal cortisol level of 28 µg /dL (N: 6.7-22.6), a basal ACTH level of 168 pg/mL (N: 0-46), midnight cortisol of 17.5 µg /dL (N <7.5) (17), midnight ACTH of 38.4 pg/mL (sample taken between 11:00 -12:00 p.m.), overnight dexamethasone suppression (ODS) cortisol of 1.18 µg/dL, and 24-hour urine free cortisol (UFC) of 504 µg/day (N <180). Repeated UFC values were 251 µg/day and 302 µg/day, respectively. A low-dose dexamethasone suppression test (LDDST) led to a cortisol level of 0.17 µg/dL and UFC of 11 µg/day (N <10). Because the cortisol level was found to be suppressed and UFC was at a borderline level, we did not feel the need to perform any further investigations. However, in the follow-up period, the patient was observed to have uncontrolled hypertension, and we were not able to attribute this finding to any other cause. Echocardiograpic examination, renal Doppler ultrasound, and renal magnetic resonance imaging (MRI) angiography were performed and revealed normal findings. The patient’s plasma renin, aldosterone, and catecholamine levels were also within normal ranges. Gadolinium-diethylenetriamine pentaacetic acid (Gd-DTPA)-enhanced MRI of the pituitary gland, abdomen and thorax was performed, and the results were normal. After stimulation with 100 µg corticotropin-releasing hormone (CRH), inferior petrosal sinus sampling (IPSS) showed a central to peripheral ratio of 4 (N <3) and lateralization to the right side with a ratio of 3.98 (Table 2). The patient was diagnosed as CD, and treatment with 1 mg/week cabergoline (given twice a week) was initiated. In the fourth month of treatment, the blood pressure returned to normal and UFC decreased to 112 and 130 µg/day in two samples. On the 17th month of treatment, the patient, receiving 1 mg/week cabergoline, was still in remission. No adverse effect of cabergoline was observed during the follow-up period.

**Patient 2:** A 15-year-old male presented with weight gain and hypertension. He weighed 107 kg and was 172 cm tall (Table 1). His blood pressure was 160/100 mmHg, BMI37 kg/m2, and pubertal development was Tanner stage V. He had a moon face and generalized obesity. Investigations were suggestive of ACTH-dependent CD: basal cortisol of 26 µg/L, basal ACTH of 135 pg/mL, ODS serum cortisol of 15.9 µg/dL, 24-hour UFC of 481 µg/day, midnight cortisol level of 14.5 µg/dL, LDDST serum cortisol level of 0.48 μg/L, and UFC of 13.1 µg /day. Gd-DTPA-enhanced MRI of the pituitary gland, abdomen, and the thorax revealed normal results. Stimulated IPSS with 100 µg CRH showed a central to peripheral ratio of 5.7 (N <3) and lateralization to the right side with a ratio of 2.8 (Table 2). The patient was diagnosed as CD, and treatment with 1 mg/week cabergoline (given twice a week) was started. At the second month of therapy, the 24-hour UFC was still 210 µg/day, and the cabergoline dose was increased to 1.5 mg/week. On the sixth month of treatment, cortisol level in ODS test was 0.9 µg /dL, and UFC was 132 and 72 µg /day in two samples. Blood pressure at this time had decreased to 130/90 mmHg. During the follow-up period, UFC was measured successively at intervals of 1-2 months. The patient was in remission by the 24th month of cabergoline treatment, administered as 1.5 mg twice weekly. No adverse effect was observed during cabergoline treatment.

## DISCUSSION

CD in childhood is a rare disorder that is generally caused by pituitary or ectopic ACTH-secreting tumors. Surgery is the principal therapeutic modality. No ACTH-secreting tumor was demonstrated by radiologic imaging methods in either of our two patients. Both patients underwent dynamic Gd-DTPA-enhanced MRI investigation using coronal sequences, and no hypothalamic or pituitary lesion was detected. However, it is known that MRI investigation can detect tumors in only about 50% of all cases. Pituitary tumors cannot be distinguished from the pituitary gland tissue, especially if they are small or have the same imaging character as the gland. In such cases, spoiled gradient sequences, which have thinner slice thicknesses and provide better tissue discrimination, should be obtained. We did not have the means to use this technique in the imaging studies of our patients. However, determination of an ACTH gradient by IPSS, on the right side in one patient (Patient 1) and on the left side in the other patient (Patient 2) suggested the presence of microadenoma which could not be detected in MRI.

Cyclic Cushing’s syndrome is a pattern of hypercortisolism in which cortisol production fluctuates. This syndrome is often associated with fluctuating symptoms and signs. Large cyclical fluctuations about every 10 days over 40 days, confirmed by repeated measurements, have been reported (18). Between exacerbations, urine and serum cortisol levels were found to be within normal ranges. Cyclic CD is very rare in childhood (19). Cyclic CD could be associated with primary pigmented nodular adrenocortical disease (20).

Serum and urine cortisol levels of our first patient were abnormal initially. The urine free cortisol was high in repeated samples. This patient had intractable hypertension and weight gain. However, because cortisol was found to be suppressed by dexamethasone and UFC was at the borderline level, we did not feel the need to perform any further investigations and did not suspect cyclic CD initially in this patient. The IPSS performed during the follow-up period helped us establish the diagnosis.

The expression and function of the D2 receptors in corticotroph pituitary tumors has been demonstrated in earlier studies. The presence of functional D2 receptors in 60% and the demonstration of effectiveness of a short-term treatment with the dopamine agonist cabergoline in normalizing ACTH and cortisol secretion in 40% of corticotroph pituitary tumors strongly support the possible therapeutic use of this drug in the management of persistent and/or recurrent CD (7). Cabergoline was described as having potential positive metabolic effects. It could lower blood pressure and improve glucose tolerance independent of its cortisol-lowering effect. Dopamine agonists lower peripheral resistance relaxing vascular wall smooth muscles, with consequent improvement of blood pressure (5).

Cabergoline is usually used as a second-line therapy in patients with unsuccessful surgical removal of a secreting tumor (5,7,16,21) . Pivonello et al (7) reported 20 patients with CD who were unsuccessfully treated by surgery. In this group, short-term (3 months) and long-term (12-24 months) effectiveness of cabergoline was evaluated. While 75% of these patients responded to cabergoline treatment in short-term, in 8 of these 20 (40%) patients, cabergoline treatment was found effective without significant side effects during a treatment period of 24 months. Vilar et al (21) also found a 25% complete response to cabergoline monotherapy in 12 CD patients who had undergone transsphenoidal surgery with unsuccessful results and who received a maximal dose of 3 mg/week of cabergoline for a period of 6 months.

Godbout et al (5) showed that short-term treatment with cabergoline in patients with CD improves cortisol secretion in 50% of subjects, with complete normalization of UFC in 36.6% of cases. Long-term follow-up during a mean period of 37 months demonstrated sustained effectiveness of cabergoline in 30% of subjects with mostly persistent or recurrent CD. Cabergoline was used as a first-line therapy in three patients of their cohort (5). One of these patients demonstrated a complete normalization of UFC after 1 month of treatment with 1 mg/week of cabergoline with regression of clinical signs following 18 months of treatment. The two other patients on first-line therapy did not respond after 3 months of treatment with 1.5 or 2 mg/week of cabergoline (5).

Both our patients were treated with the dopamine agonist cabergoline and both showed a complete normalization of UFC after 6 months of treatment with doses of 1 and 1.5 mg/week, respectively. Both patients are still in remission with improved clinical symptoms and normal UFC at the end of 17th and 24th months, respectively.

In adults, cabergoline was also used in CD caused by macroadenomas. Beside the control of aberrant ACTH secretion, significant reduction and stabilization of macroadenoma volume was demonstrated by cabergoline treatment in patients with CD (14,22).

Although data about cabergoline as first-line therapy in CD are very limited, the safety profile of this product, its relative efficacy, and its surgery-sparing potential appear to warrant the need for prospective studies on its long-term efficacy in larger cohorts of patients with CD. An extensive literature search revealed only a few reports of adolescent patients who were treated with cabergoline because of persistent CD after surgery and radiotherapy (9,16). Cabergoline treatment in childhood was reported by Gopal et al (9). Their patient was a 12-year-old boy who had persistent CD and who had undergone surgery and radiotherapy. The authors suggested that cabergoline was a useful option for inducing remission in uncured CD patients. Lila et al (16) reported 18 uncured CD patients, 4 of whom were adolescents treated with cabergoline. They found that cabergoline was an effective therapy in terms of LDDST and/or midnight cortisol results in 28% of patients with uncured CD. Our patients had a quite good response to treatment with no adverse effects.

In conclusion, cabergoline appears to be a particularly interesting first-line therapeutic option for patients with radiologically nondetectable CD. Further studies on a larger population of patients are necessary to establish the definitive results of cabergoline treatment.

## Figures and Tables

**Table 1:**
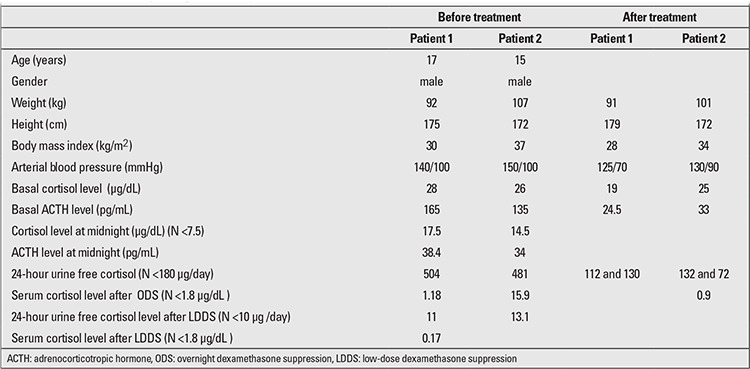
Clinical and laboratory findings in the two patients

**Table 2:**
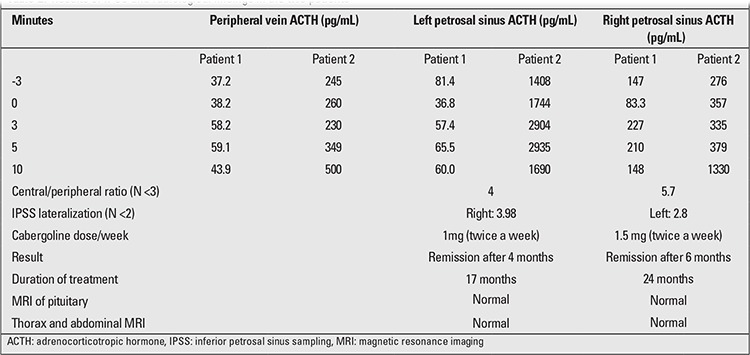
Results of IPSS and radiological findings in the two patients
